# Health care access and utilization among adult cancer survivors: Results from the National Institutes of Health “All of Us” Research Program

**DOI:** 10.1002/cam4.3924

**Published:** 2021-05-03

**Authors:** Steven S. Coughlin, Jie Chen, Jorge E. Cortes

**Affiliations:** ^1^ Department of Population Health Sciences Medical College of Georgia Augusta University Augusta GA USA; ^2^ Institute of Public and Preventive Health Augusta University Augusta GA USA; ^3^ Department of Medicine Medical College of Georgia Augusta University Augusta GA USA; ^4^ Georgia Cancer Center Augusta University Augusta GA USA

**Keywords:** cancer, cancer survivors, health care access, health care utilization

## Abstract

**Background:**

Many cancer survivors face financial difficulties that prevent them from receiving appropriate health care. Racial/ethnic disparities in receipt of health care have been reported among cancer survivors, but recent data for important racial/ethnic subgroups of the US population are lacking.

**Methods:**

To learn more about barriers to healthcare access faced by cancer survivors, we analyzed data from the NIH “All of Us” Research Program. Data were analyzed about demographic factors and other personal characteristics, personal medical history of cancer, healthcare utilization, and access to care.

**Results:**

As of November 2020, a total of 5426 participants had a history of cancer (excluding skin cancer). About 88.2% were non‐Hispanic White; 3.9% were Black, African American, or African; 1.3% were Asian; 4.1% were Hispanic, Latino, or Spanish; and 1.2% reported more than one race. Just over one‐half had an annual income of $75,000 or greater. The majority of the participants (71.7%) were college graduates or had an advanced degree. About 47.0%% had private health insurance, 41.0% had Medicare, 6.0% had Medicaid, and the remainder had military, Veterans Affairs, other insurance, or no health insurance. Frequently cited reasons for delayed care in the past 12 months were “had to pay out of pocket for some or all of the procedures,” “deductible was too high/or could not afford the deductible,” “couldn't afford the copay,” “couldn't get time off work,” and “were nervous about seeing a health care provider.”

**Discussion:**

A minority of cancer survivors who participated in the NIH “All of Us” Program had difficulty paying for health care in the past 12 months. Of particular concern are minorities such as African American and Hispanic cancer survivors along with those who are low income.

## Abstract 2

Many cancer survivors face financial problems that prevent them from receiving timely and recommended health care. A minority of cancer survivors who participated in the NIH “All of Us” Research Program had difficulty paying for health care in the past 12 months. Of particular concern are minorities such as African American and Hispanic cancer survivors along with those who are low income.

## 1 INTRODUCTION

Cancer survival is improving over time as a result of screening and improved treatment.^1^ An estimated 16.9 million cancer survivors were alive on January 1, 2019, in the US Cancer survivors often have life‐long health care needs that include surveillance for recurrent or secondary primary malignancies, preventive care, and management of chronic conditions and cancer treatment‐related morbidity.^2^, ^3^

Healthcare utilization among cancer survivors varies by age, race, income, insurance status, tumor type, and stage.^4^ Many cancer survivors face financial difficulties that prevent them from receiving appropriate health care.^5^, ^6^ The cost of cancer care is an important barrier to health care access, particularly among those who are racial minorities, have lower income, or are uninsured.^5^, ^7^ Studies have shown that uninsured cancer survivors are more likely to avoid care and prescription medications due to cost.^8^, ^9^, ^10^ Lack of insurance in cancer survivors has been associated with forgoing care and poorer clinical outcomes.^11^

Racial/ethnic disparities in receipt of health care have been documented among cancer survivors.^3^, ^9^, ^10^, ^12^, ^13^ In an analysis of data from the 2006–2010 National Health Interview Survey, Palmer et al.^3^ found that non‐Hispanic Whites reported receiving more health care than did Hispanics and African Americans. In a study of adolescent and young adult cancer survivors ages 15–39 years, Keegan et al.^9^ found that among those who had no cancer‐related medical visits in the previous year, a greater frequency were of non‐Hispanic Black or American Indian/Alaska Native and Hispanic race/ethnicity compared to those who received care. In a similar study by Kirchoff et al.^12^ Hispanic cancer survivors had the lowest frequency of insurance among the survivors, and two thirds of this racial/ethnic group had no routine medical care in the previous year. American Indian/Alaska Native cancer survivors also experience challenges in accessing health care.^14^, ^15^

There is also a correlation between age and receiving health care among cancer survivors.^12^, ^16^ Health care utilization among older cancer survivors is influenced by the high frequency of comorbid diseases and treatment complications.^16^ On the other hand, younger cancer survivors may be especially likely to forgo health care because of cost barriers. Kirchhoff et al.^12^ found that more than one‐fifth of adolescent and young adult survivors did not have a healthcare provider. More than two‐thirds of uninsured survivors received no routine health care.^12^ Compared to older cancer survivors, younger patients who are in their 20s are often less affluent and more likely to be without health insurance. Younger cancer patients, particularly those who are ethnic minority and low income, often have challenges such as delays in care.^17^, ^18^

To learn more about barriers to healthcare access among cancer survivors, we analyzed data from the National Institutes of Health (NIH) “All of Us” Research Program. Survey data were analyzed about demographic factors and other personal characteristics, personal medical history of cancer, healthcare utilization, and access to care. Of particular interest were racial and ethnic disparities in health care. We hypothesized that individuals who are Hispanic or African American would be less likely to have received health care and experience greater barriers to care compared to non‐Hispanic Whites. The current study adds to the published literature by providing greater information about reasons for delayed care among cancer survivors. The study also adds additional information about racial disparities in access to care among cancer survivors.

## 2 METHODS

The NIH “All of Us” Research Program is open to all eligible adults who live in the United States. Participants can sign up directly through the NIH “All of Us” Research Program website or through participating health care provider organizations. Although the data are not population‐based, they reflect the diversity of cancer survivors in the US population in terms of race, ethnicity, age, gender, health status, and other factors. In the current study, cancer survivors were defined as research participants who had been diagnosed with cancer; the sample included both individuals who were undergoing cancer treatment and those who had already completed primary therapy for the disease. Individuals with skin cancer were excluded from the analysis because their cancer survivorship care needs are often relatively minor. The “All of Us” Research Program is approved by the NIH Institutional Review Board (IRB). Participants sign an informed consent document according to the Declaration of Helsinki authorizing the collection of their data. De‐identified data from individual participants are available to approved researchers.

Survey data were analyzed about demographic factors and other personal characteristics (age, sex, race, Hispanic ethnicity, educational attainment, marital status, health care insurance, employment status, annual household income, and number of people in household); personal medical history of cancer; general health; and health care utilization. Under personal medical history, the participants were asked, “Has a doctor or health care provider ever told you that you have or had any of the following cancers?” and a list of 21 cancer types followed including “Other cancer.” Under health care utilization, the participants were asked a number of questions as summarized in Table 1.

**TABLE 1:** Questions about healthcare access and utilization, NIH “All of Us” Research Program

“During the past 12 months, were you told by a health care provider or doctor's office that they did not accept your health care coverage?”
“In regard to your health insurance or health care coverage, how does it compare to a year ago? Better, worse, or about the same.”
“Is there a place that you usually go to when you are sick or need advice about your health?”
“What kind of place do you go to most often? Doctor's office clinic or health center, urgent care or minute clinic, hospital emergency room, some other place, or don't go to one place most often.”
“About how long has it been since you last saw or talked to a doctor or other health care provider about your own health?”
“In the past 12 months, have you seen or talked to a general doctor who treats a variety of illnesses?”
“What is the total number of general doctor visits you made in the last 12 months.”
“In the past 12 months, have you seen or talked to a nurse practitioner, physician assistant, or midwife?”
“What is the total number of nurse practitioner, physician assistant, or midwife visits you made in the last 12 months.”
“In the past 12 months, have you seen or talked to a doctor who specializes in women's health (an obstetrician/gynecologist)?”
“What is the total number of obstetrician/gynecologist visits you made in the last 12 months.”
“In the past 12 months, have you seen or talked to a doctor who specializes in a particular medical disease or problem?”
“What is the total number of visits you made to a medical doctor who specializes in a particular medical disease or problem that you made in the last 12 months?”
“Have you delated getting care for any of the following reasons in the past 12 months? Didn't have transportation, you live in a rural area where distance to the health care provider is too far, you were nervous about seeing a health care provider, couldn't get time off work, couldn't get child care, you provide care to an adult and could not leave him/her, couldn't afford the copay, your deductible was too high/or could not afford the deductible, or you had to pay out of pocket for some or all of the procedure.”
“During the past 12 months, was there any time when you needed any of the following, but didn't get it because you couldn't afford it? Prescription medicines, to see a regular doctor or general health provider, to see a specialist, or follow up care.
“If you get sick or have an accident, how worried are you that you will be able to pay your medical bills? Very worried, somewhat worried, not at all worried.”
“During the past 12 months, were any of the following true for you? You skipped medications to save money, you took less medicine to save money, you delayed filling a prescription to save money, you delayed filling a prescription to save money, you asked your doctor for a lower cost medication to save money, you bought prescription drugs from another country to save money, or you used alternative therapies to save money.”

In summarizing the results, any group with less than 20 participants was combined with a relevant group to satisfy the safe data sharing policy of “All of Us”. After frequencies and cross‐tabulations of the data were completed, multivariate logistic regression methods were used to examine self‐reported delayed receipt of health care according to age, sex, race, Hispanic ethnicity, education, annual household income from all sources, number of people in household, marital status, and health insurance. Ninety‐five percent confidence intervals were obtained for adjusted odds ratios. Levels of statistical significance were determined using Wald chi‐square tests and log‐likelihood ratio tests. The goodness‐of‐fit of each model was examined using the log‐likelihood ratio test.

## 3 RESULTS

As of November 2020, a total of 5426 participants in the NIH “All of Us” Research Program had a history of cancer, excluding skin cancer (Table 2). Their mean age was 67.6 years (standard deviation = 12.0). Overall, 64.3% were women and 35.7% were men. About 88.2% were non‐Hispanic White; 3.9% were Black, African American, or African; 1.3% were Asian; 4.1% were Hispanic, Latino, or Spanish; and 1.2% reported more than one race. There was wide variation in self‐reported annual income. Just over one‐half had an annual income of $75,000 or greater. Over two‐thirds were married or living with a partner. The majority of the participants (71.7%) were college graduates or had an advanced degree. About 47.0% had private health insurance, 41.0% had Medicare, 6.0% had Medicaid, and the remainder had military, Veterans Affairs, other insurance, or no health insurance. Table 3 shows the type(s) of cancer reported by the participants. The most frequent cancer types included breast, prostate, and colorectal cancer, which represented 27.4%, 14.3%, and 5.2% of the cancers, respectively. Only 2.6% of the cancer survivors had lung cancer, which has a lower survival rate.

**TABLE 2:** Personal characteristics of cancer survivors, NIH “All of Us” Research Program

Characteristic	Frequency (%)
Age (years) mean =67.6 (SD =12.0)	
Sex	
Male	1919 (35.7)
Female	3457 (64.3)
Race (categories not mutually exclusive)	
White, Non‐Hispanic	4827 (88.2)
Black, African American, or African	216 (3.9)
Asian	69 (1.3)
Hispanic, Latino, or Spanish	227 (4.1)
Other	64 (1.2)
More than one race	67 (1.2)
Annual household Income	
<$10,000	125 (2.3)
$10,000–$24,999	354 (6.6)
$25,000–$34,999	334 (6.2)
$35,000–$49,999	476 (8.9)
$50,000–$74,999	825 (15.4)
$75,000–$99,999	719 (13.4)
$100,000–$149,999	967 (18.0)
$150,000–$199,999	475 (8.9)
$200,000 or more	655 (12.2)
Prefer not to answer	433 (8.1)
Number of people in household	
1	1155 (21.7)
2	2936 (55.2)
3	670 (12.6)
4	333 (6.3)
5+	226 (4.2)
Marital status	
Married	3360 (62.5)
Living with partner	218 (4.1)
Never married	580 (10.8)
Widowed	381 (7.1)
Separated	59 (1.1)
Divorced	781 (14.5)
Education	
Less than HS	44 (0.8)
HS or equivalent	345 (6.4)
Some college	1168 (21.6)
College graduate or advanced degree	3838 (71.7)
Health insurance	
Medicare	1925 (41.0)
Medicaid	282 (6.0)
Military	93 (2.0)
Veterans affairs	109 (2.3)
Purchased	551 (11.7)
Employer or union	1658 (35.3)
Other	82 (1.7)
None	—

**TABLE 3:** Cancer‐related medical history among cancer survivors, NIH “All of Us” Research Program

Condition	Frequency (%)
Cancer (cancer types not mutually exclusive)	
Bladder cancer	177 (2.9)
Blood or soft tissue cancer	435 (7.1)
Bone cancer	70 (1.1)
Brain cancer	56 (0.9)
Breast cancer	1693 (27.4)
Cervical cancer	352 (5.7)
Colon cancer/rectal cancer	319 (5.2)
Endocrine cancer	51 (0.8)
Endometrial cancer	198 (3.2)
Esophageal cancer	41 (0.7)
Eye cancer	29 (0.5)
Head and neck cancer	157 (2.5)
Kidney cancer	212 (3.4)
Lung cancer	162 (2.6)
Ovarian cancer	145 (2.4)
Pancreatic cancer	48 (0.8)
Prostate cancer	881 (14.3)
Stomach cancer	32 (0.5)
Thyroid cancer	361 (5.9)
Other type	749 (12.1)

Table 4 summarizes health care access and utilization among the cancer survivors. About 9.7% were told by a health care provider or doctor's office that they did not accept their health care coverage during the past 12 months. About 8.9% reported that their health insurance or health care coverage was worse than a year ago, and 8.0% reported that it was better. Only 2.2% reported no place that they usually go to when they are sick or need advice. The majority of the participants (93.5%) had seen or talked to a general doctor or a specialist (83.4%) in the past 12 months. The most common reasons for delayed care in the past 12 months were “had to pay out of pocket for some or all of the procedures,” “deductible was too high/or could not afford the deductible,” “couldn't afford the copay,” “couldn't get time off work,” and “were nervous about seeing a health care provider.” For a minority of the participants (3.4% to 10.0%), there was a time during the past 12 months when they needed prescription medications, a regular doctor or general health provider, a specialist, or follow‐up care, but did not get it because they could not afford it.

**TABLE 4:** Health care access and utilization among cancer survivors, NIH “All of Us” Research Program

Condition	Frequency (%)
During the past 12 months, were you told by a health care provider or doctor's office that they did not accept your health care coverage	
Yes	552 (9.7)
No	4839 (90.3)
In regard to your health insurance or health care coverage, how does it compare to a year ago?	
Better	423 (8.0)
Worse	474 (8.9)
About the same	4419 (83.1)
Is there a place that you usually go to when you are sick or need advice about your health?	
Yes	4088 (77.0)
There is no place	116 (2.2)
There is more than one place	1107 (20.8)
What kind of place do you go to most often?	
Doctor's office, clinic or health center	4960 (94.4)
Urgent care or minute clinic	150 (2.9)
Hospital emergency room	34 (0.6)
Some other place	36 (0.7)
Don't go to one place most often	74 (1.4)
About how long has it been since you last saw or talked to a doctor or other health care provider about your own health?	
Never	—
6 months or less	5031 (94.1)
More than 6 months, but not more than 1 year ago	275 (5.1)
More than 1 year, but not more than 2 years ago	38 (0.7)
More than 2 years, but not more than 5 years ago	—
More than 5 years ago	—
In the past 12 months, have you seen or talked to a general doctor who treats a variety of illnesses?	
Yes	4963 (93.5)
No	344 (6.5)
What is the total number of general doctor visits you made in the last 12 months?	
1	711 (14.5)
2–3	1793 (36.5)
4–5	973 (19.8)
6–7	457 (9.3)
8–9	231 (4.7)
10–12	243 (4.9)
13–15	111 (2.3)
16 or more	396 (8.1)
In the past 12 months, have you seen or talked to a nurse practitioner, physician assistant, or midwife?	
Yes	3385 (66.2)
No	1726 (33.8)
What is the total number of nurse practitioner, physician assistant, or midwife visits you made in the last 12 months?	
1	1288 (39.3)
2–3	1296 (39.5)
4–5	349 (10.6)
6–7	120 (3.7)
8–9	63 (1.9)
10–12	75 (2.3)
13 or more	89 (2.7)
In the past 12 months, have you seen or talked to a doctor who specializes in women's health (an obstetrician/gynecologist)?	
Yes	1416 (42.6)
No	1908 (57.4)
What is the total number of obstetrician/gynecologist visits you made in the last 12 months?	
1	843 (60.2)
2–3	373 (26.6)
4–5	97 (6.9)
6–7	26 (1.9)
8 or more	61 (4.4)
In the past 12 months, have you seen or talked to a doctor who specializes in a particular medical disease or problem?	
Yes	4384 (83.4)
No	871 (16.6)
What is the total number of visits you made to a medical doctor who specializes in a particular medical disease or problem that you made in the last 12 months?	
1	585 (13.5)
2–3	1610 (37.0)
4–5	937 (21.6)
6–7	416 (9.6)
8–9	236 (5.4)
10–12	225 (5.2)
13–15	88 (2.0)
16 or more	250 (5.8)
Have you delayed getting care for any of the following reasons in the past 12 months?	
Didn't have transportation	
Yes	227 (4.3)
No	5081 (95.7)
You live in a rural area where distance to the health care provider is too far	
Yes	125 (2.4)
No	5152 (97.6)
You were nervous about seeing a health care provider	
Yes	419 (7.9)
No	4860 (92.1)
Couldn't get time off work	
Yes	283 (5.4)
No	4963 (94.6)
Couldn't get child care	
Yes	41 (0.8)
No	5141 (99.2)
You provide care to an adult and could not leave him/her	
Yes	77 (1.5)
No	5172 (98.5)
Couldn't afford the copay	
Yes	304 (5.8)
No	4945 (94.2)
Your deductible was too high/or could not afford the deductible	
Yes	331 (6.3)
No	4893 (93.7)
You had to pay out of pocket for some or all of the procedure	
Yes	691 (13.1)
No	4584 (86.9)
During the past 12 months, was there any time when you needed any of the following, but didn't get it because you couldn't afford it?	
Prescription medicines	
Yes	536 (10.0)
No	4806 (90.0)
To see a regular doctor or general health provider	
Yes	182 (3.4)
No	5155 (96.6)
To see a specialist	
Yes	324 (6.1)
No	5010 (93.9)
Follow‐up care	
Yes	253 (4.7)
No	5075 (95.3)
If you get sick or have an accident, how worried are you that you will be able to pay your medical bills?	
Very worried	377 (7.1)
Somewhat worried	1813 (34.3)
Not at all worried	3097 (58.6)
During the past 12 months, were any of the following true for you?	
You skipped medications to save money	
Yes	339 (6.3)
No	5023 (93.7)
You took less medicine to save money	
Yes	392 (7.3)
No	4955 (92.7)
You delayed filling a prescription to save money	
Yes	565 (10.6)
No	4784 (89.4)
You asked your doctor for a lower cost medication to save money	
Yes	1264 (23.7)
No	4064 (76.3)
You bought prescription drugs from another country to save money	
Yes	274 (5.1)
No	5067 (94.9)
You used alternative therapies to save money	
Yes	319 (6.0)
No	5036 (94.0)

We then analyzed the factors that most influenced the delays in receiving medical care in the previous 12 months among these cancer survivors. Table 5 shows the results of a multivariate logistic regression for such predictors. After adjusting for other variables in the model, cancer survivors who were younger, women, or had lower income were more likely to report delays in receiving medical care in the previous 12 months, along with those who had only completed some college.

**TABLE 5:** Multivariate logistic regression results for predictors of delayed receipt of medical care in the past 12 months among cancer survivors, NIH “All of Us” Research Program

Covariate	Beta coefficient	Odds ratio	95% confidence interval	*p*‐value
Age	−0.06010	0.94	0.93, 0.95	0.00
Sex				
Male	−0.18981	0.83	0.69, 0.99	0.043
Female		1.00	—	
Race				
Black/African American	−0.16547	0.85	0.57, 1.26	
White		1.00	—	
Asian	−0.30028	0.74	0.33, 1.50	
Other	0.40185	1.49	0.91, 2.44	NS
Ethnicity				
Hispanic/Latino		1.00	—	NS
Not hispanic	−0.36636	0.69	0.42, 1.14	
Household income				
<$10,000	1.13693	3.12	1.75, 5.55	
$10,000–$24,999	1.32659	3.77	2.57, 5.53	
$25,000–$34,999	0.79384	2.21	1.52, 3.22	
$35,000–$49,999	0.91603	2.50	1.80, 3.46	
$50,000–$74,999	0.50691	1.66	1.25, 2.21	
$75,000–$99,999	0.27477	1.32	0.98, 1.76	
$100,000–$149,999		1.00	—	
$150,000–$199,999	0.02537	1.03	0.71, 1.47	
$200,000 or more	−1.29033	0.75	0.53, 1.05	0.00
No. of people in household				
1	−0.11392	0.89	0.64, 1.81	
2	−0.20618	0.78	0.63, 1.05	
3		1.00	—	
4	−0.04277	0.96	0.66, 1.38	
5	0.30474	1.36	0.79, 2.30	
6+	0.50467	1.66	0.95, 2.88	0.45
Marital status				
Married		1.00	—	
Living with partner	0.14338	1.15	0.76, 1.72	
Never married	−0.08901	0.91	0.65, 1.28	
Widowed	−0.51344	0.60	0.39, 0.90	
Separated	0.04512	1.05	0.49, 2.16	
Divorced	0.05948	1.00		NS
Education				
Less than HS	−0.16346	0.85	0.39, 1.79	
HS or equivalent	0.12699	1.14	0.82, 1.55	
College graduate or advanced degree		1.00	—	NS
Health insurance				
Medicare		1.00	—	
Medicaid	−0.03238	0.97	0.67, 1.39	
Military	−0.65526	0.52	0.23, 1.04	
Veterans affairs	0.52045	1.68	1.00, 2.75	
Employer/union	0.11145	1.12	0.90, 1.39	
Purchased	0.10951	1.12	0.84, 1.48	
Other	0.27673	1.32	0.73, 2.31	NS

## 4 DISCUSSION

The results of this study indicate that a majority of cancer survivors who are participants in the NIH “All of Us” Research Program have seen a doctor or a specialist in the past 12 months, but that a significant number of participants delayed care due to out‐of‐pocket health care expenses such as deductibles or copayments. Approximately 3.4% to 10.0% of the participants reported no health care or follow‐up care because they could not afford it, even though the participants were more educated and had higher annual incomes, on average, than the US population as a whole. It is likely that these percentages are much higher among cancer survivors in the general population, and particularly among minorities and other populations suffering significant health disparities.

Cancer survivors who have completed primary therapy for the disease often have complex health care needs that include surveillance for recurrent disease or second primary malignancies, and the management of cancer‐related morbidities.^2^ Appropriate care during the post‐initial treatment survivorship phase includes preventive care visits and screening for cancer recurrence.^2^ In the current study, a high percentage (93.5%) of cancer survivors had seen a general doctor in the past 12 months and 83.4% had seen a specialist such as an oncologist. Previous studies of cancer survivors have shown high (92% to 97%) rates of adherence to routine primary care visits in the previous year.^19^, ^20^ In a study of survivors of non‐Hodgkin lymphoma, 82% of the patients reported visits to oncologists.^21^

Racial/ethnic disparities in general health care utilization are frequent.^3^ Minorities are less likely to use health care services than are non‐Hispanic Whites.^3^ Factors that contribute to these disparities include low income and lack of health insurance.^22^, ^23^, ^24^ In the current study, African Americans and Hispanics were not significantly more likely to report delaying getting care in the past 12 months than non‐Hispanic Whites. Only a small percentage of participants reported that they lacked health insurance. This is likely an underrepresentation of this problem in the general population as individuals included in this database are a self‐selected population; less educated and lower income patients are underrepresented and more likely to suffer from the factors most limiting of access to health care in general. In an analysis of data from the National Health Interview survey, Palmer et al.^3^ found that older African American and Hispanic cancer survivors were about twice as likely as non‐Hispanic Whites to not see a specialist. Similar racial/ethnic differences in health care use have been reported in Surveillance Epidemiology and End Results‐Medicare studies.^25^, ^26^

Our study has limitations that need to be considered when interpreting the results. The source of data is not population‐based and, as mentioned, the participants were relatively educated and most had health insurance. Therefore, the results may not be generalizable to all cancer survivors in the US population. We compared findings from the current study with results obtained by Zeng et al.^27^ in their analysis of data on cancer survivors from the 2013–2017 National Health Interview Survey (NHIS). In contrast to the current study, the NHIS is representative of the US population. In the study by Zeng et al.,^27^ 52.6%–65.4% of the sample of cancer survivors were women compared to 64.3% of our sample from the NIH “All of Us” Research Program. In the study by Zeng et al.,^27^ 57.9%–60% of the cancer survivors were married compared to 62.5% of the current sample. In the study by Zeng et al.,^27^ the percentages of the cancer survivors who were White, Black/African American, or Hispanic were 77.9%–83.8%, 7.3%–8.0%, and 5.0%–9.2%, respectively, compared to 88.2%, 3.9%, and 4.1% of the current sample. Thus, in the current study, there was a higher percentage of non‐Hispanic Whites, and lower percentages of Blacks and Hispanics. The current study also included more cancer survivors who were married and fewer survivors who were uninsured. A further issue is that the numbers of participants who are American Indian, Alaska Native, or Pacific Islander were too small for separate estimates. Still, the results highlight the issues that cancer survivors face when dealing with long‐term care and trying to manage their long‐term medical needs associated with cancer survivorship.

In summary, a minority of cancer survivors who participated in the NIH “All of Us” Research Program had difficulty paying for health care in the past 12 months. However, these issues are magnified among minorities such as African American and Hispanic and those with lower income.

## CONFLICT OF INTEREST

The authors have no conflicts of interest to disclose.

## ETHICAL STATEMENT

The study was approved by the NIH Institutional Review Board. The current study was approved by the Augusta University IRB and was based on “All of Us” survey data version 3. The IRB determined that the study does not meet the definition of human subjects research as there are no personal identifiers in the dataset available to researchers.

## Data Availability

The data are available from the NIH “All of Us” Research Program.
